# ARMS技术联合Taqman探针检测100例非小细胞肺癌*EGFR*基因突变

**DOI:** 10.3779/j.issn.1009-3419.2013.01.05

**Published:** 2013-01-20

**Authors:** 静 赵, 金银 赵, 肖 赵, 唯军 陈, 巍 钟, 力 张, 龙芸 李, 孟昭 王

**Affiliations:** 1 100730 北京，中国医学科学院，北京协和医学院，北京协和医院呼吸内科 Department of Respiratory Medicine, Peking Union Medical College Hospital, Peking Union Medical College and Chinese Academy of Medical Sciences, Beijing 100730, China; 2 100029 北京，中国科学院北京基因组研究所 Key Laboratory of Genome Sciences and Information, Beijing Institute of Genomics, Chinese Academy of Sciences, Beijing 100029, China

**Keywords:** 扩增阻滞突变系统, Taqman探针, 表皮生长因子受体, 突变检测, 肺肿瘤, Amplification refractory mutation system, Taqman probe, Epidermal growth factor receptor, Mutation detection, Lung neoplasms

## Abstract

**背景与目的:**

表皮生长因子受体（epidermal growth factor receptor, *EGFR*）基因突变是决定表皮生长因子受体酪氨酸激酶抑制剂（EGFR tyrosine kinase inhibitor, EGFR-TKI）疗效最重要的预测因子，对*EGFR*基因突变进行检测，对指导患者个体化治疗具有重要意义。*EGFR*基因突变检测方法有很多，每种方法各有优缺点，本研究拟采用扩增阻滞突变系统（amplification refractory mutation system, ARMS）技术与Taqman探针相结合的方法，建立一种能快速、敏感及特异检测非小细胞肺癌*EGFR*基因突变的方法。

**方法:**

首先，应用Primer Premier 5.0软件在*EGFR*基因E746_A750和L858R处设计ARMS引物及Taqman水解探针。然后，以包含E746_A750缺失和L858R点突变的质粒为研究对象，进一步分析所建立方法的灵敏度、敏感性以及特异性。最后，用所建立的ARMS-Taqman法检测100例非小细胞肺癌（non-small cell lung cancer, NSCLC）临床标本。

**结果:**

在无背景DNA干扰的情况下，ARMS-Taqman法检测灵敏度可达10 copies。对于检测敏感性，在500 copies/μL野生型基因背景下，其敏感性达1%;在5, 000 copies/μl野生型基因背景下，其敏感性高达0.1%-0.5%。对于检测特异性，以正常人白细胞DNA为研究对象，21 L858R突变存在一定程度的非特异性扩增，但其最小ΔCt高达14.89，而19 Del未见非特异性扩增。对100份临床标本进行检测，19 Del 21例，21 L858R 18例，总突变率为39.0%。

**结论:**

我们所构建的ARMS-Taqman法是一种快速、简便以及具有较高灵敏度和特异性的*EGFR*基因突变检测方法，值得在临床上进一步推广和验证。

## Abstract (en)

**Background and objective:**

Epidermal growth factor receptor (*EGFR*) gene mutation is the most important predictor of the efficiency of EGFR-tyrosine kinase inhibitors in the treatment of non-small cell lung cancer (NSCLC). The detection of *EGFR* gene mutations can guide individual therapies for NSCLC. Numerous methods are used to detect *EGFR* gene mutation and each method has different features. This study aims to establish a real-time polymerase chain reaction (PCR) method for the detection of *EGFR* gene mutations using amplification refractory mutation system (ARMS) specific primers and Taqman fluorescence probes.

**Methods:**

ARMS specific primers for the two *EGFR* gene mutations (E746_A750 and L858R) and Taqman fluorescence probes for the detection of the target sequence were carefully designed by the Primer Premier 5.0 software. Then, using the recombinants containing E746_A750 and L858R mutations as the study objects, we further analyzed the sensitivity and lower limit of this method, and then determined the cutoff ΔCt value to evaluate specific or non-specific amplification. A total of 100 clinical samples were collected and used to detect the *EGFR* gene mutations using this method.

**Results:**

The lower limit of this method for the detection of *EGFR* gene mutation was 10 copies if no interference of wild-type *EGFR* gene or background DNA existed. Regarding the method sensitivity, the detection resolution was as high as 1% and 0.1%-0.5% in the background of 500 and 5, 000 copies/μL wild-type *EGFR* gene, respectively. Regarding the method specificity, non-specific amplifications were found when it was used to detect 21 L858R mutations in leukocyte DNA samples from healthy volunteers. However, the minimal ΔCt value was 14.48. Non-specific amplifications were not found when detecting 19 Del mutations. Among the 100 clinical samples, 39 mutations were detected (19 Del and 21 L858R were 21 and 18, respectively) using this method. The total mutation rate was 39.0%.

**Conclusion:**

The proposed ARMS-TaqMan real-time PCR method for the detection of 19 Del and 21 L858R mutations in *EGFR* gene was rapid, simple, sensitive, specific, and applicable in the clinical setting.

表皮生长因子受体（epidermal growth factor receptor, EGFR）是一种跨膜受体酪氨酸激酶，该区域的激活对非小细胞肺癌（non-small cell lung cancer, NSCLC）的增殖、生长相关信号传递具有重要意义。大量研究^[1-3]^表明，*EGFR*基因突变状态是决定EGFR酪氨酸激酶抑制剂（tyrosine kinase inhibitor, TKI）疗效最重要的预测因子，故在晚期NSCLC中检测*EGFR*基因突变状态至关重要，是决定患者能否一线应用EGFR-TKI的先决条件。*EGFR*基因突变检测方法有很多，其中Scorpions ARMS法具有检测灵敏度高、操作简便、结果容易判读、省时等诸多优点，在一些大型临床研究^[2]^中被广泛采用，但其高昂的检测费用并不适合我国国情。为此，我们自主设计了*EGFR*基因常见突变的ARMS引物，并联合Taqman探针技术建立了一种快速、简便、经济以及灵敏的检测方法，并取得了满意效果，特此汇报。

## 1 材料与方法

### 1.1 ARMS引物及Taqman探针的设计

在美国国立生物技术信息中心（National Center of Biotechnology Information, NCBI）数据库下载*EGFR*全基因序列。EGFR Exon 19 E746_A750 del和Exon 21 L858R突变信息分别为2235_2249 del 15和2573 T > G。应用Primer Premier 5.0软件在E746_A750和L858R处设计ARMS引物，其长度约22个碱基，GC含量为40%-60%，Tm值为58 ℃-60 ℃，扩增片段大小为80 bp-150 bp。Taqman探针长度为26个-30个碱基，GC含量为40%-60%，Tm值为68 ℃-70 ℃。另在EGFR Exon 2上设计引物和探针，作为内参。以携带Exon 19 E746_A750缺失突变和Exon 21 L858R点突变的质粒标准品（由中国科学院北京基因组研究所赠送）为研究对象，筛选和确立最佳引物和探针。

荧光定量PCR反应体系如下（20 μL反应体系）：2 ×Taqman Universal PCR Mix 10 μL，上下游引物（10 μM）各0.5 μL，探针（10 μM）0.4 μL，DNA模板（2 ng/μL-20 ng/μL）2 μL，纯水定容至20 μL。PCR反应程序：50 ℃、5 min; 95 ℃预变性10 min; 40个循环：95 ℃、15 s、60 ℃、45 s（收集荧光）。

### 1.2 灵敏性试验

将Exon 19 E746_A750 del和Exon 21 L858R质粒标准品按10^0^ copies/μL、1×10^1^ copies/μL、1×10^2^ copies/μL、1×10^3^ copies/μL、1×10^4^ copies/μL、1×10^5^ copies/μL进行梯度稀释，按1.1所建立的方法，进行灵敏性试验，每个梯度设3个重复孔。

### 1.3 敏感性试验

#### 1.3.1 19外显子缺失突变敏感性试验

将Exon 19 E746_A750 del突变型质粒分别加入到500 copies/μL和5, 000 copies/μL的Exon 19野生型质粒（由中国科学院北京基因组研究所赠送）中，制成突变率（突变型基因与野生型基因拷贝数百分比）依次为10%、5%、1%、0.5%、0.1%、0%的样本，按1.1所建立的方法进行检测，以确定其检测敏感性。

#### 1.3.2 21外显子点突变敏感性试验

将Exon 21 L858R突变型质粒分别加入到500 copies/μL和5, 000 copies/μL的Exon 21野生型质粒（由中国科学院北京基因组研究所赠送）中，制成突变率依次为10%、5%、1%、0.5%、0.1%、0%的样本，按1.1所建立的方法进行检测，以确定其检测敏感性。

### 1.4 特异性试验及cutoff值确定

以10份正常人白细胞DNA为研究对象，浓度范围1 ng/μL-50 ng/μL，进行6次重复性试验，统计每次出现的非特异性扩增Ct值及样本的内参Ct值，计算出ΔCt值（ΔCt=Ct_非特异_-Ct_内参_）。依据公式cutoff ΔCt=平均ΔCt-3×Sd-3，计算出cutoff ΔCt。

### 1.5 100份NSCLC组织标本的检测

#### 1.5.1 标本收集、DNA提取

NSCLC标本来自2008年12月-2010年12月北京协和医院呼吸内科肺癌病房收治，且经临床、影像以及病理诊断为非小细胞肺癌患者，共100例，其中男性53例，女性47例; 腺癌71例（包括细支气管肺泡癌5例），鳞癌22例，其它7例（包括腺鳞癌1例，未定型6例）; 既往采用Scorpions ARMS法检测过的标本29例。每例患者提供石蜡切片5张。采用离心柱法提取石蜡切片DNA（使用商品化Tiangen组织样本基因组DNA提取试剂盒）。最后，使用紫外分光光度计测定DNA浓度，调整DNA浓度于2 ng/μL-20 ng/μL之间，冻存于-20 ℃冰箱备用。

#### 1.5.2 ARMS联合Taqman探针技术检测NSCLC组织*EGFR*基因突变

##### 1.5.2.1 阳性对照品制备

以提取的20 ng/μL正常人全基因组DNA为背景，加入Exon 19 E746_A750 del和Exon 21 L858R突变型质粒，制成1:1的突变阳性对照品。

##### 1.5.2.2 临床样本的检测

按照1.1所建立的ARMS-Taqman PCR反应体系及反应程序对所收集的临床样本进行检测，每一份样本均设立一个阴性对照样本（以纯水代替）和一个阳性对照样本。

计算出每个样本的ΔCt（ΔCt=Ct_突变反应_-Ct_内参_），依据1.4所建立的cutoff ΔCt值判断结果。若ΔCt值小于cutoff ΔCt值，则为阳性样本; 若ΔCt值大于cutoff ΔCt值，则为阴性样本或超出检测范围。

对ARMS-Taqman PCR检测阳性的标本均进行测序验证，测序由上海生工完成。

## 2 结果

### 2.1 ARMS引物及Taqman探针

以2外显子为内参。表 1列出了2外显子内参引物，19外显子缺失突变ARMS引物、21外显子点突变ARMS引物，以及用于各自扩增产物检测的Taqman探针序列。

**1:** 检测*EGFR*基因Exon 19 E746_A750 del和Exon 21 L858R突变所设计的ARMS引物和Taqman探针序列 Sequences of ARMS primers and Taqman probes designed for the detection of Exon 19 E746_A750 del and Exon 21 L858R mutations in *EGFR* gene

	Primers (5’-3’)	Probes (5’-3’)
Exon 2 Internal control	F: TGCCAAGGCACGAGTAACAAG R: TCCAAATTCCCAAGGACCAC	FAM-TCTCAGCCTCCAGAGG ATGTTCAATAACT-BHQ1
Exon 19 E746_A750 del	F: AATTCCCGTCGCTATCAAAAC R: ACCCCCACACAGCAAAGC	FAM-CCAACAAGGAAATCCT CGATGTGAGTTTCTG-BHQ1
Exon 21 L858R	F: AAGATCACAGATTTTGTGCG R: CAGCCTGGTCCCTGGTGT	FAM-TTCTTTCTCTTCCGCAC CCAGCAGT-BHQ1

### 2.2 ARMS-Taqman PCR反应体系检测灵敏度结果

对*EGFR*基因突变检测的2个反应（19 Del和21 L858R），ARMS-Taqman PCR方法最低可检测至1×10^1^ copies/μL。图 1、图 2分别显示19 Del和21 L858R检测反应。

**1:**
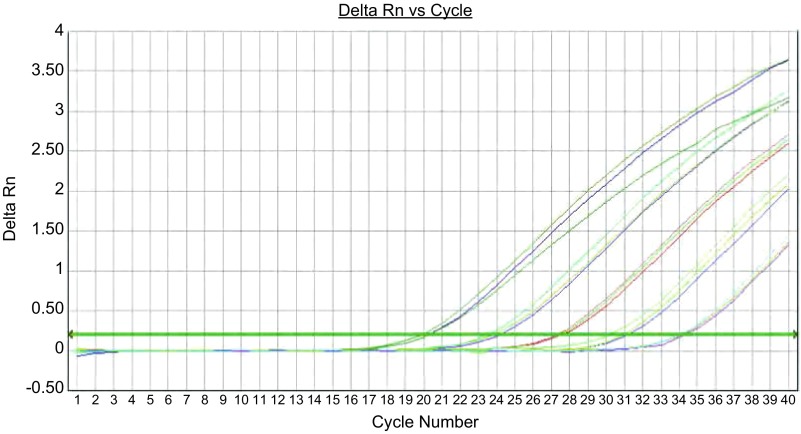
19 Del检测曲线，19 Del突变型质粒浓度梯度依次为：1×10^5^ copies/μL、1×10^4^ copies/μL、1×10^3^ copies/μL、1×10^2^ copies/μL、1×10^1^ copies/μL。 The amplification curves of 19 Del mutation (the concentrations of mutant plasmid was 1×10^5^ copies/μL, 1×10^4^ copies/μL, 1×10^3^ copies/μL, 1×10^2^ copies/μL and 1×10^1^ copies/μL, respectively).

**2:**
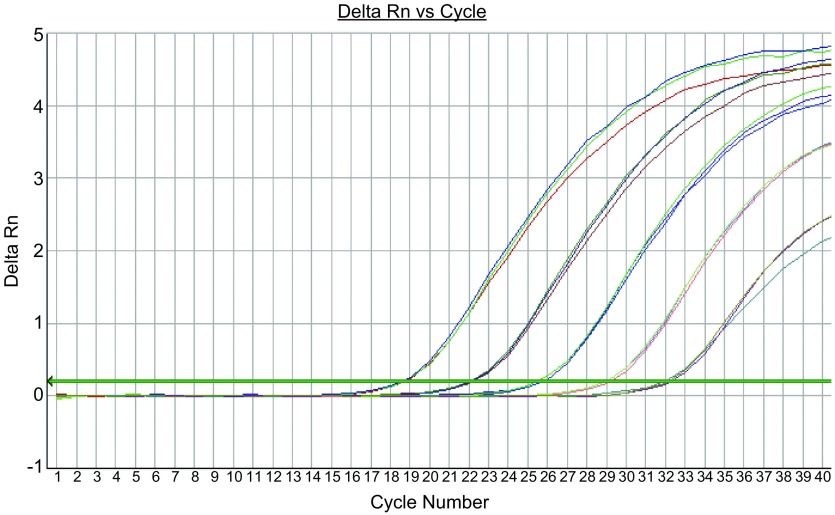
21 L858R检测曲线，21 L858R突变型质粒浓度梯度依次为：1×10^5^ copies/μL、1×10^4^ copies/μL、1×10^3^ copies/μL、1×10^2^ copies/μL、1×10^1^ copies/μL。 The amplification curves of 21 L858R mutation (the concentrations of mutant plasmid was 1×10^5^ copies/μL, 1×10^4^ copies/μL, 1×10^3^ copies/μL, 1×10^2^ copies/μL and 1×10^1^ copies/μL, respectively).

### 2.3 ARMS-Taqman PCR反应体系检测敏感性结果

对于19 Del突变，如图 3，在500 copies/μL的野生型质粒背景下，可检出突变率为1%;如图 4，在5, 000 copies/μL的野生型质粒背景下，可检出突变率为0.5%。对于21 L858R，如图 5，在500 copies/μL的野生型质粒背景下，可检出突变率为1%;如图 6，在5, 000 copies/μL的野生型质粒背景下，可检出突变率为0.1%。

**3:**
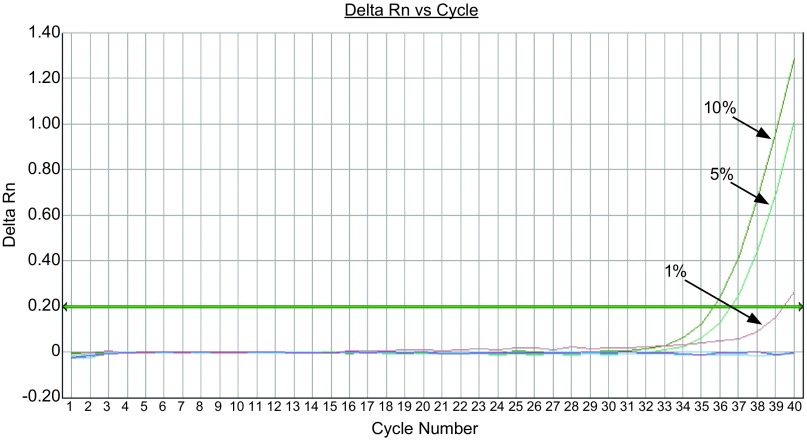
19 Del在500 copies/μL野生型质粒背景下，依次存在10%、5%、1%、0.5%、0.1%、0%的突变率，ARMS-Taqman PCR可检出含1%突变率样本。 The 19 Del mutation amplification curves for different concentrations of mutant plasmids (under the background of 500 copies/μL wild-type plasmids) showed the detection resolution was 1%.

**4:**
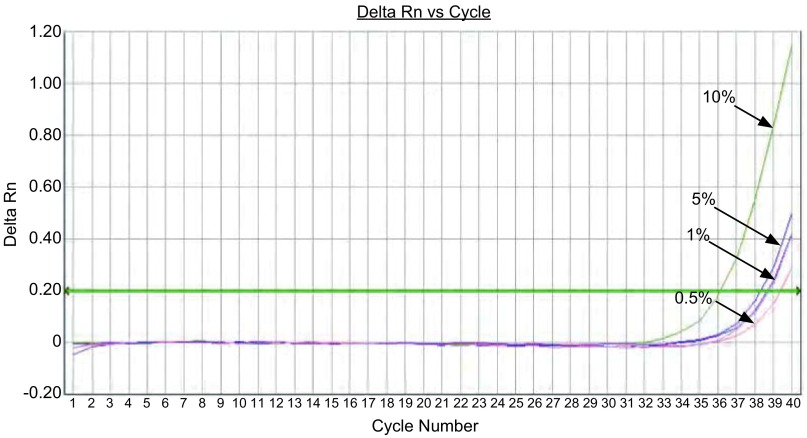
19 Del，在5, 000 copies/μL野生型质粒背景下，依次存在10%、5%、1%、0.5%、0.1%、0%的突变率，ARMS-Taqman PCR可检出含0.5%突变率样本。 The 19 Del mutation amplification curves for different concentrations of mutant plasmids (under the background of 5, 000 copies/μL wild-type plasmids) showed the detection resolution was 0.5%.

**5:**
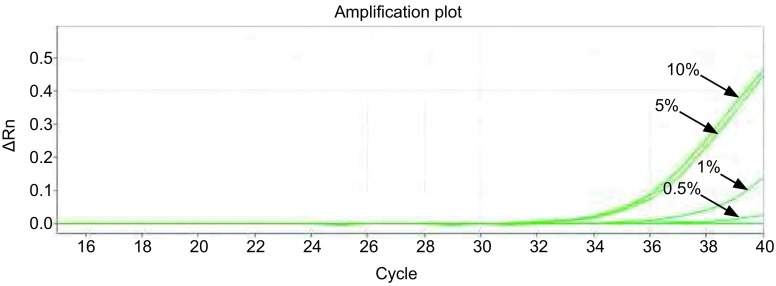
21 L858R，在500 copies/μL野生型质粒背景下，依次存在10%、5%、1%、0.5%、0.1%、0%的突变率，ARMS-Taqman PCR可检出含1%突变率样本。 The 21 L858R mutation amplification curves for different concentrations of mutant plasmids (under the background of 500 copies/μL wild-type plasmids) showed the detection resolution was 1%.

**6:**
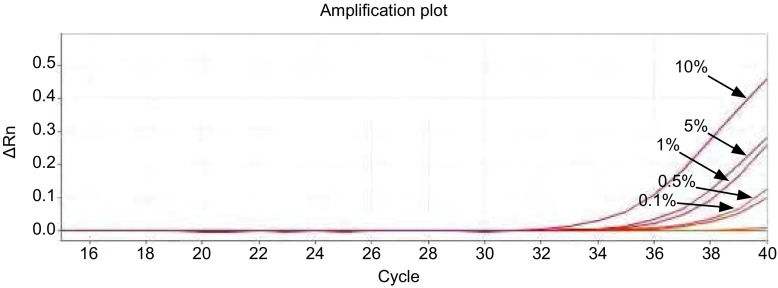
21 L858R，在5, 000 copies/μL野生型质粒背景下，依次存在10%、5%、1%、0.5%、0.1%、0%的突变率，ARMS-Taqman PCR可检出含0.1%突变率样本。 The 21 L858R mutation amplification curves for different concentrations of mutant plasmids (under the background of 5c000 copies/μL wild-type plasmids) showed the detection resolution was 0.1%.

### 2.4 ARMS-Taqman PCR反应体系cutoff ΔCt值

对于检测特异性，如表 2所示，19 Del未见非特异性扩增，cutoff ΔCt设定为10。而21 L858R突变存在一定程度的非特异性扩增（6/60），根据计算公式，cutoff ΔCt为10。

**2:** Cutoff ΔCt值统计结果 The summary of cutoff ΔCt values

Mutation	Mean ΔCt±SD	Minimal ΔCt	Maximal ΔCt	The occurrence frequency of non-specific amplification	cutoff ΔCt
19 Del	\*	\	17.24	0	10
21 L858R	15.53±0.47	14.89	15.95	6/60	10
^*^ means the non-specific amplification.					

### 2.5 100例NSCLC临床样本检测结果

100例NSCLC组织标本，采用ARMS-Taqman PCR检测*EGFR*基因突变，结果显示，19 Del 21例，21 L858R 18例，总突变率为39.0%（39/100）。从*EGFR*基因突变分布看，19 Del占51.2%（21/39），21 L858R占43.9%（18/39）。

对ARMS-Taqman检测阳性标本进行测序验证，结果显示，测序结果与ARMS-Taqman荧光检测结果完全一致。图 7、图 8分别显示19 Del和21 L858R ARMS-Taqman检测结果与测序结果。

**7:**
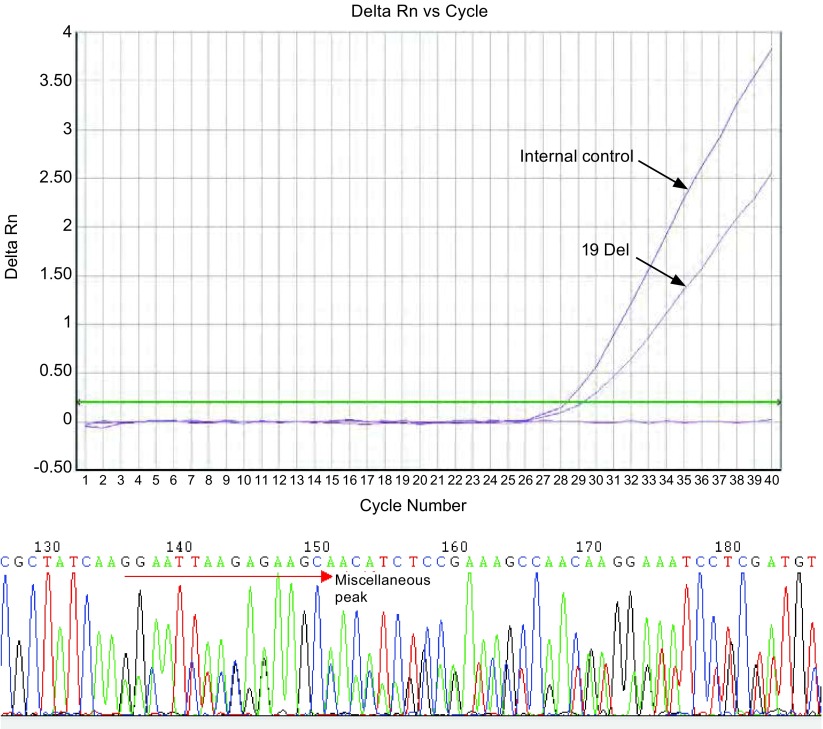
样本1 Exon 19 E746_A750 Deletion突变：荧光检测与测序结果 Exon 19 E746_A750 Deletion mutation (sample 1): the results of ARMS-Taqman and sequencing

**8:**
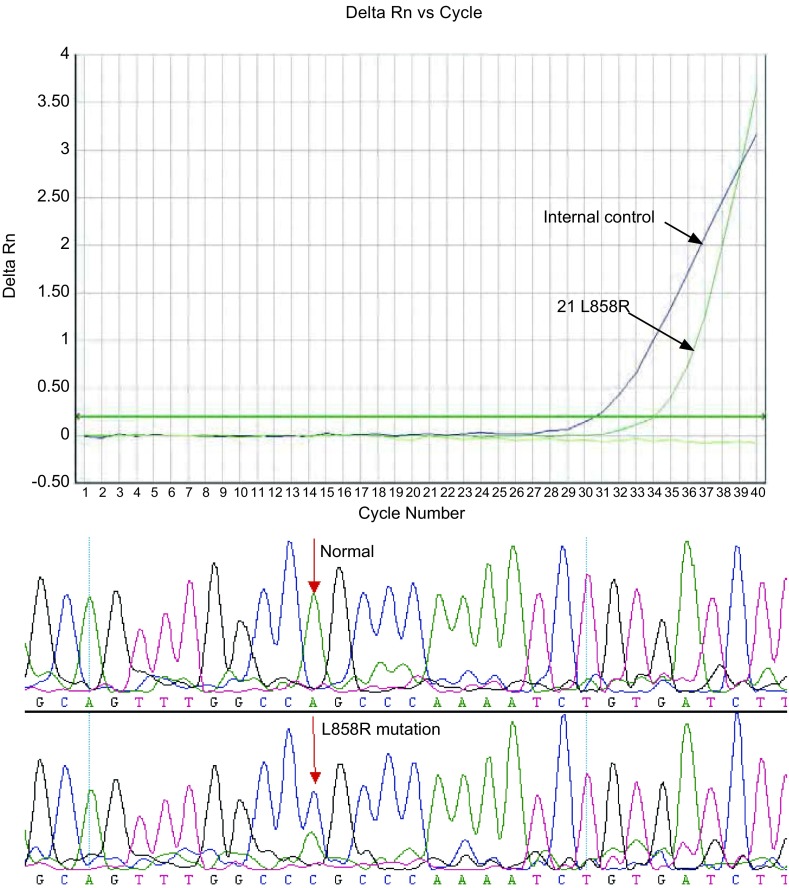
样本2 Exon 21 L858R突变：荧光检测与测序结果 Exon 21 L858R mutation (sample 2): the results of ARMS-Taqman and sequencing

100例NSCLC标本中，有29例标本既往曾采用Scorpions ARMS法进行过*EGFR*基因突变检测。在这29例标本中，Scorpions ARMS法检出19 Del 3例，占10.3%（3/29），21 L858R 6例，占20.7%（6/29），总突变率为31.0%（9/29）。对这29例标本，ARMS-Taqman法检出19 Del 3例，占10.3%（3/29），21 L858R 5例，占17.2%（5/29），总突变率为27.6%（8/29）。两种方法19 Del突变检出一致率为93.1%，Kappa=0.627;21 L858R突变检出一致率为96.6%，Kappa=0.890。

## 3 讨论

多项临床研究^[1-6]^表明：与标准化疗相比，具有*EGFR*基因突变的NSCLC患者使用EGFR-TKI，具有更高的客观缓解率和PFS，同时患者生活质量更佳。鉴于此，早在2011版NCCN NSCLC指南就明确指出，对于Ⅳ期非鳞NSCLC患者，应先行*EGFR*基因突变检测，如果存在*EGFR*基因突变，治疗上优先推荐EGFR-TKI。可见，*EGFR*基因突变的检测对指导患者个体化治疗方案的制定具有重要意义。

目前，已经报道的*EGFR*基因突变类型大约有60种^[7]^，其中29种突变最为常见，包括19种19 Del、3种20 Ins、21 L858R、21 L861Q、20 T790M、20 S768I、18 G719A、18 G719S和18 G719C，它们约占*EGFR*基因突变类型的99%^[8]^，其它*EGFR*基因突变类型罕见，而且和EGFR-TKI疗效之间的关系并不确切，因此并未在临床上指导EGFR-TKI的使用，故仅对这29种常见*EGFR*基因突变进行检测，足以满足临床所需。

关于*EGFR*基因突变检测的方法有很多，各国学者对此都进行了大量研究。已经报道的方法包括^[9]^：直接测序法、DHPLC、PCR-SSCP/RFLP、Scorpions ARMS、Taqman PCR、ME-PCR等。这些方法各有优缺点，其中在临床和科研中较为常用的方法为直接测序法和Scorpions ARMS法。直接测序法检测能力有限，其检测敏感性约20%左右，而且步骤复杂、费时费力，但该方法的优点是能够发现一些新的未知突变。Scorpions ARMS方法是英国QIAGEN公司开发的一种商业化试剂盒，其即是针对上述常见29种*EGFR*基因突变进行检测，该方法是将分子信标（Scorpions探针、蝎形探针）与特异性的ARMS引物相结合创造出来的，ARMS引物3’端设计在突变位点，最后一个碱基与突变碱基配对，只有引物3’末端完全配对时，才能正常扩增，当引物3’末端发生错配时，不能有效的扩增。当引物与突变模板结合并延伸出相应的产物后，引物5’端的探针部分自身双链解开并与扩增产物结合，而使分子信标两端的荧光基团和淬灭基团分离产生荧光。该方法敏感性高达1%，同时还具有操作简便、结果判读容易、省时等多种优点^[10]^。但是，因其能在一次PCR反应中同时检测这29种*EGFR*基因突变，众多ARMS引物和Scorpions探针使得检测费用昂贵，约4, 000元/例，不适合我国NSCLC患者的常规检测和筛查。

本研究中所建立的Taqman-ARMS检测方法亦是针对这29种*EGFR*基因突变位点，通过自主设计的ARMS引物，同时使用普通的Taqman水解探针代替Scorpions探针，使得检测成本大大降低，约300元/例。出于对知识产权的保护，我们在此仅报道了针对19 Del和21 L858R位点设计的ARMS引物和Taqman探针。在无野生型基因以及背景DNA干扰的情况下，本研究所建立的ARMS-Taqman PCR方法检测灵敏度可达10 copies，敏感性达0.1%-1%，同时具有较高的特异性。在对100例NSCLC临床标本的检测中，本研究所创立的ARMS-Taqman方法检出*EGFR*基因突变率为39%（39/100），这与文献^[11-13]^中报道的亚裔患者*EGFR*基因具有30%-50%左右的突变率的结果一致。从*EGFR*基因突变分布看，19 Del占53.85%（21/39），21 L858R占46.15%（18/39），这亦与文献^[8]^中报道的*EGFR*基因突变分布情况较为吻合。在与Scorpions ARMS的比较研究中，两种方法具有较高的检测一致率。

综上，本研究所构建的ARMS-Taqman法是一种快速、简便以及具有较高灵敏度和特异性的*EGFR*基因突变检测方法，价格低廉，值得在临床上进一步推广和验证。
